# Chronotyping glaucoma patients with the Munich ChronoType Questionnaire: A case-control study

**DOI:** 10.1371/journal.pone.0214046

**Published:** 2019-03-28

**Authors:** Ronald A. J. M. Bierings, Marijke C. M. Gordijn, Nomdo M. Jansonius

**Affiliations:** 1 Department of Ophthalmology, University of Groningen, University Medical Center Groningen, Groningen, the Netherlands; 2 Chronobiology Unit, Groningen Institute for Evolutionary Life Sciences, University of Groningen, Groningen, the Netherlands; 3 Chrono@Work B.V., Groningen, the Netherlands; CNRS, University of Strasbourg, FRANCE

## Abstract

**Purpose:**

The circadian clock is entrained to light by the intrinsically photosensitive retinal ganglion cells. Loss of these cells in glaucoma, an eye disease with loss of retinal ganglion cells as its key feature, might thus result in a change in chronotype. We aimed to compare the chronotype between glaucoma patients and healthy subjects.

**Methods:**

We sent the Munich ChronoType Questionnaire to 221 glaucoma patients (response rate 81%); controls (primary control group) were primarily their spouses. After exclusion of shift workers and participants who woke-up due to an alarm clock on days off, 159 glaucoma patients (88 early, 21 moderate, 23 severe) and 163 controls remained. We calculated chronotype as the mid-sleep on days off, corrected for workweek accumulated sleep debt (MSF_sc_). We compared means and variances between groups using Welch’s tests and F-tests, respectively. A secondary control group was recruited from participants in a citizen-science project (n = 17073) who completed an online questionnaire. A resampling method was applied to enable an age- and gender- matched comparison with the glaucoma patients.

**Results:**

Compared to the primary control group, glaucoma did not affect the mean MSF_sc_ (controls 3:47; early, moderate, and severe glaucoma 3:40, 3:45, and 3:33, respectively [P = 0.62]). Chronotype variability seemed to increase with increasing disease severity (severe glaucoma versus controls: P = 0.023). The mean MSF_sc_ of the secondary control group was 3:50 (95% confidence interval 3:48 to 3:52); significantly later than that of the glaucoma patients (3:40; P = 0.024). Mean MSF_sc_ did not differ significantly between the control groups (P = 0.42).

**Conclusions:**

No clear changes were found in the chronotype as determined by sleep phase in patients with glaucoma, especially not in early and moderate glaucoma. In severe glaucoma, chronotype variability seems to increase, possibly alongside a small advancement.

## Introduction

Glaucoma is a chronic and progressive eye disease characterized by loss of retinal ganglion cells (RGCs) and subsequent visual field loss. Among the different types of RGCs, the intrinsically photosensitive retinal ganglion cells (ipRGCs) express melanopsin and are held responsible for nonvisual responses to light, such as the pupillary light reflex [1–3] and the entrainment of the circadian clock to light [4–8]. Output of the ipRGCs is transmitted to the suprachiasmatic nucleus, the circadian clock that drives rhythms with a period of approximately 24 hours in physiology, sleep-wake behaviour, and cognitive performance [9–11]. In absence of light cues, the circadian system will lose its synchronisation to the Earth’s 24-hour light/dark cycle, the Zeitgeber [12,13], and this leads to a mismatch between endogenous rhythms and the sleep-wake cycle. Hence, loss of ipRGC function in glaucoma might result in circadian misalignment and thus disturb the sleep quality and pattern of glaucoma patients [14]. Interestingly, the light-induced melatonin suppression, as one of the nonvisual responses to light, was found to be affected in patients with advanced glaucoma [15–17], and glaucoma patients often do report a lower sleep quality [18–21]. It is controversial, however, if the latter is related to RGC damage or to psychological factors [22].

Human circadian phase can be described by means of the chronotype of an individual. The chronotype of an individual can be defined as the midpoint between sleep onset and wake-up time on days off [23] corrected for sleep on working days (Mid-Sleep on Free days, Sleep debt on work days Corrected; MSF_sc_) [24]. The chronotype as defined by sleep phase should be considered as a marker of circadian phase, and it has been shown to correlate well with other circadian phase parameters such as the start of melatonin production [24–27]. Functional damage of ipRGCs might lead to misalignment of the circadian clock to light resulting in either freerunning patterns of sleep and wakefulness, or to modulations of the direct effects of light on sleep and wakefulness [4,28]. The intrinsic period of the circadian clock in humans differs between individuals and is on average a little bit longer than 24 hours [13,29–31]. The entrained phase of the circadian pacemaker is dependent on the intrinsic period showing a later sleep phase with longer intrinsic period [25,32–35]. Consequently, damage to the ipRGCs in glaucoma might result in a delay of the mean MSF_sc_ and an increase in sleep phase variability. A delay and an increase in variability in activity onsets has indeed been found in animal studies to glaucoma [36,37]. More variability in waking time was also observed in a diverse group of young subjects with an optic nerve disease, including some patients with glaucoma [38]. Intriguingly, studies to the entrained circadian phase of glaucoma patients appear to be completely lacking.

The aim of this study was to compare chronotype as a measure of circadian phase between glaucoma patients and healthy subjects. For this purpose, we performed a questionnaire study with the Munich ChronoType Questionnaire (MCTQ) and determined the chronotype distribution amongst a large group of glaucoma patients and controls.

## Methods

### Study population and data acquisition

The MCTQ was sent by mail to 221 glaucoma patients (cases) with open-angle glaucoma (primary or related to pseudoexfoliation or pigment dispersion). Patients were participants in the Groningen Longitudinal Glaucoma Study (GLGS). The GLGS is an observational cohort study conducted in the University Medical Center Groningen [39]. We approached those participants who were still visiting our clinic, were followed with standard automated perimetry (SAP; Humphrey field analyzer [HFA] 30–2 SITA; Carl Zeiss Meditec AG, Jena, Germany), and had a reproducible visual field defect on SAP in at least one eye, defined as a scotoma according to the LTG-P criterion [40] or a glaucoma hemifield test ‘outside normal limits’. For descriptive statistics, the patients were stratified into early, moderate, or severe glaucoma, using the mean deviation (MD) value of the better eye (eye with the higher MD value) [41–46] corresponding to the most recent visual field test. As cut-off points between the strata we employed -6 and -12 dB. For the classification, we used the most recent visual field test result.

Two questionnaires were sent to each patient; they were asked to complete one questionnaire and to give the other to their spouse, neighbor, friend, etc. (no consanguinity), who served as control [47]. Patients and controls were explicitly asked to fill in the questionnaire independently. As the number of returned patient questionnaires exceeded the number of control questionnaires (in 30% only the patient questionnaire was returned), additional controls were recruited from a recent case-control studies conducted in our department [48]. Controls were asked to confirm that they (1) did not have relatives with high eye pressure or glaucoma and (2) did not receive regular checkups by an ophthalmologist for high eye pressure or glaucoma. In this way we assured a glaucoma prevalence of <1% amongst the controls [49].

A secondary control group was obtained by taking an age- and gender-matched sample from 17073 subjects who participated in an internet-based citizen-science project. Details of the study protocol and the results for the first 5055 subjects have been described before [50]. From these subjects, only their age and gender was known.

The ethics board of the University Medical Center Groningen (UMCG) approved the study protocol (METc 2014.338). All participants provided written informed consent. The study followed the tenets of the Declaration of Helsinki.

### Data analysis

Shift workers and participants who woke-up due to an alarm clock on days off were excluded from the analyses. The study population was described using descriptive statistics. Univariable comparisons between cases and controls (from the primary control group) were made with a t-test or Mann-Whitney test, depending on the distribution, for continuous variables; for proportions we used a Chi-square test with Yates correction.

For questions regarding bedtime information on days off (Q1-Q8; see Results section), the mean and standard deviation (SD) were determined for glaucoma patients and controls (from the primary control group). Sleep onset was calculated as the sum of the point of time to get ready to fall asleep, and the length of time needed to actually fall asleep (Q2 and Q3). The sleep duration was defined as the difference between the calculated sleep onset and the wake-up time (Q4). The mid-sleep on days off (MSF) was defined as the midpoint between sleep onset and wake-up time. When the sleep duration during the workweek was shorter compared to that of days off, we corrected the MSF (MSF_sc_) for workweek accumulated sleep debt [24]. We compared means with a Welch’s t-test (unlike the default t-test, this test allows for unequal variances) and distributions with an F-test. For MSF_sc_, we also performed a comparison after stratification to disease severity (early glaucoma: MD of better eye above -6 dB; moderate glaucoma: MD between -6 and -12 dB; severe glaucoma: MD below -12 dB) using a Welch F-test (an alternative to one-way analysis of variance (ANOVA) that does not assume the variances to be equal) to compare means and F-tests to compare variances. If significant differences between disease severity strata were found, we also performed a trend analysis. Analyzes were performed using R (version 3.4.2; R Foundation for Statistical Computing, Vienna, Austria). A P value of 0.05 or less was considered statistically significant.

From the citizen-science project participants (n = 17073), we selected all participants with age 42 (age of youngest glaucoma patient) and above (n = 4571; median age 51, range 42–100, interquartile range [IQR] 46–57)). From this subset, we took an age- and gender-matched sample (matched to the glaucoma patients) using propensity score matching in R (matchit with method = "nearest", discard = "both", and ratio = 1). This sampling was repeated 30 times, yielding a mean MSF_sc_ with corresponding confidence interval (CI).

## Results

We retrieved 178 questionnaires from 221 glaucoma patients (response rate 81%) and 182 questionnaires from controls. After exclusion of shift workers and participants who woke-up due to an alarm clock on days off, 159 glaucoma patients and 163 controls remained. Table 1 shows the characteristics of the study population. The group of glaucoma patients was older and consisted of fewer females, compared to the controls. Most of the patients had early glaucoma (63%); about one-third had either moderate (16%) or severe (21%) glaucoma in the better eye.

**Table 1:** Characteristics of the study population.

	Glaucoma patients (n = 159)	Controls (n = 163)	P value	Missing (%)
Age (year; mean [SD])	72.2 (10.0)	65.9 (10.5)	<0.001	0.0
Gender, female, n (%)	77 (48%)	105 (64%)	0.005	0.0
BMI (kg/m^2^; mean [SD])	26.2 (4.7)	26.1 (4.9)	0.81	5.3
Smoker, n (%)	15 (9.4%)	16 (9.8%)	1.0	0.0
Working days per week (days; median [IQR])	0 (0 to 0)	0 (0 to 3)	0.004	5.3
HFA MD of the better eye (dB; median [IQR])	-4.5 (-10.7 to -1.9)	NA	NA	0.0

SD = standard deviation; BMI = body mass index; IQR = interquartile range; HFA MD = mean deviation of Humphrey Field Analyzer; NA = not applicable.

**Fig 1:**
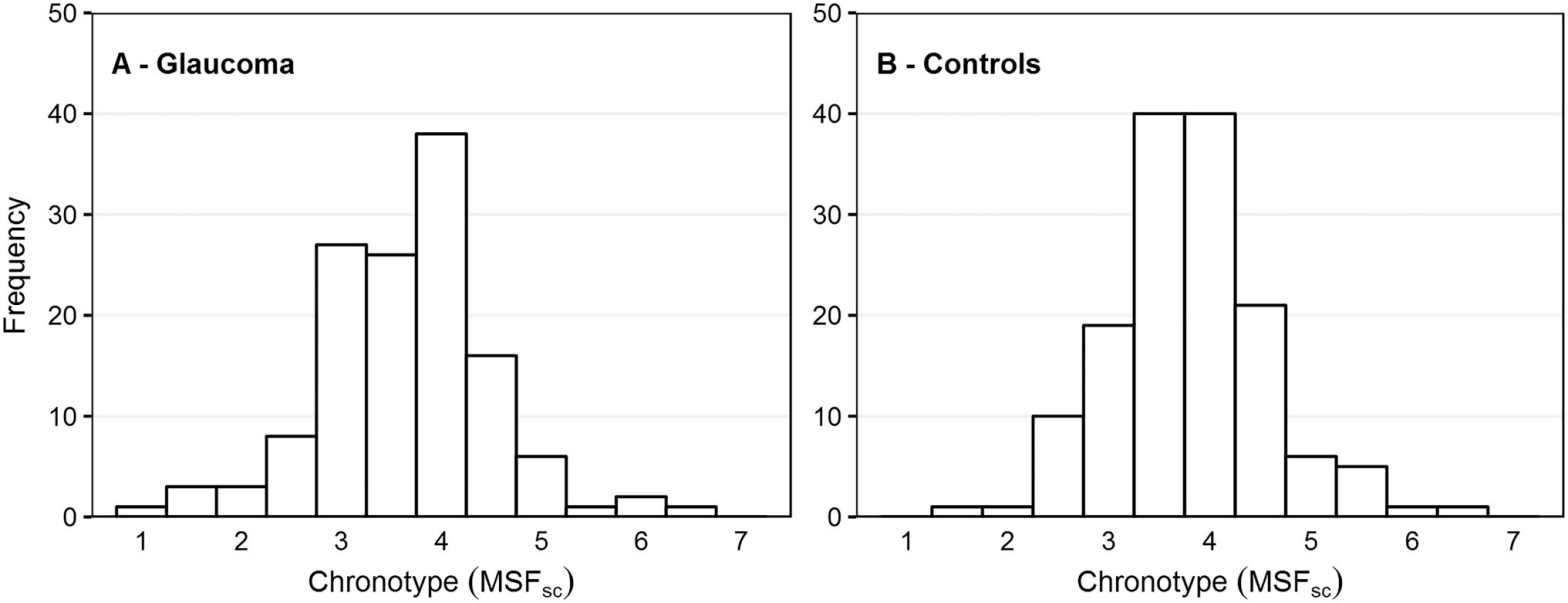
Histogram with frequency as a function of chronotype (MSFsc) for patients with glaucoma (A) and controls (B).

Table 2 presents the results from the MCTQ (A) and the corresponding calculated variables (B). We used the 24-hour clock notation for questions regarding time (23:30 is half past eleven p.m.) and duration (0:30 is 30 minutes, i.e., 0.5 hours). The original questions (Table 2A) revealed no major differences in average sleep timing parameters between the groups; however, for bedtime (Q1), time to get ready to fall asleep (Q2), sleep latency (Q3), minutes to get up after waking (Q5), and hours spent outside (Q8), the variability appeared to be larger in the glaucoma patients than in the controls, although only for Q5 a Bonferroni corrected P value of 0.006 (0.05/8) was reached. Fig 1 presents the distribution of chronotypes (MSF_sc_). The mean and distribution of the MSF_sc_ were not significantly different between glaucoma patients and controls (Table 2B; P = 0.21 for mean and P = 0.15 for variability). Table 3 shows the corresponding results after stratification to disease severity. Because of missing data (reported in the last column of Table 2), the total number of glaucoma patients and controls in Table 3 differs slightly from the total numbers in Tables 1 and 2. The mean MSF_sc_ did not differ between the groups (P = 0.62). The variability of MSF_sc_ was significantly larger for the patients with severe glaucoma compared to the controls (P = 0.023); the variability of MSF_sc_ showed a non-significant trend to increase with disease severity (P = 0.057).

**Table 2:** MCTQ derived bedtime information on days off.

	Glaucoma patients (n = 159) Mean (SD)	Controls (n = 163) Mean (SD)	P value For Mean (SD)	Missing (%)
**A. Questionnaire results**				
Q1. I go to bed at … o’clock	23:24 (0:55)	23:27 (0:46)	0.56 (0.013)	5.6
Q2. I actually get ready to fall asleep at … o’clock	23:42 (0:53)	23:48 (0:45)	0.36 (0.025)	7.5
Q3. I need … minutes to fall asleep	0:16 (0:15)	0:16 (0:17)	0.71 (0.036)	9.6
Q4. I wake up at … o’clock	7:25 (1:11)	7:37 (1:07)	0.13 (0.23)	7.1
Q5. After … minutes I get up	0:29 (0:39)	0:25 (0:27)	0.24 (<0.001)	6.8
Q6. After … minutes I feel awake	0:07 (0:13)	0:07 (0:14)	0.81 (0.29)	7.5
Q7. The quality of my nightrest (1–10)	6.7 (1.7)	6.9 (1.6)	0.37 (0.29)	4.3
Q8. Hours spent outside	2:50(2:02)	2:48 (1:41)	0.84 (0.013)	6.8
**B. Calculated variables**				
Sleep onset	23:58 (0:56)	00:04 (0:49)	0.32 (0.046)	11.2
Sleep duration	7:28 (1:12)	7:33 (1:08)	0.58 (0.28)	12.1
MSF_sc_	3:40 (0:53)	3:47 (0:48)	0.21* (0.15)	13.7

The 24-hour clock notation is used for questions regarding time (23:30 is half past eleven p.m.) and duration (0:30 is 30 minutes, i.e., 0.5 hours).

* = age- and gender-adjusted P value 0.91.

**Table 3:** MSF_sc_ mean and standard deviation as a function of disease severity.

	n	MSF_sc_ mean	P value*	MSF_sc_ SD	P value^†^
Controls	146	3:47	0.62	0:48	
Early glaucoma	88	3:40		0:49	0.40
Moderate glaucoma	21	3:45		0:55	0.20
Severe glaucoma	23	3:33		1:05	0.023

SD = standard deviation;

* = Welch F-test;

^†^ = significance of MSF_sc_ SD compared to the controls.

The mean MSF_sc_ of the secondary control group was 3:50 (95% CI 3:48 to 3:52). Compared to this control group, the mean MSF_sc_ of the glaucoma patients was significantly earlier (difference 0:10; P = 0.024). Mean MSF_sc_ did not differ between the control groups (P = 0.42).

## Discussion

Glaucoma appears not to have a substantial effect on the mean chronotype (MSF_sc_). Possibly, MSFsc is slightly advanced in glaucoma patients and—related to that—has an increased its variability.

The chronotype as a function of age in healthy subjects has been investigated in a large open study of around 25,000 subjects from Germany and Switzerland. In agreement with our study, the MSF_sc_ in subjects older than 50 years of age was between 3 and 4 AM, with a standard deviation of 1 hour [24]. Although chronotype was not assessed in glaucoma before, some studies that included glaucoma patients presented data on sleep timing. In agreement with our findings, they showed a general similarity between glaucoma patients and controls [18,22,51]. Albeit no differences in sleep timing, a lower sleep efficiency (the amount of actual sleep during the night) and quality have been reported in glaucoma patients [18–22]. Of note, the previous studies did not analyze working days and days off separately. Since the sleep pattern on work days significantly differs from the sleep pattern on days off, the comparison to our study is limited [23].

A limitation of the current study is that the glaucoma patients and controls (from the primary control group) significantly differed with respect to age and gender. However, the change of MSF_sc_ with age above 45 years of age is small, and gender differences also appear only significant below 45 years of age. Therefore, age and gender differences between our groups are presumably hardly relevant [24]. To confirm this, we adjusted the MSF_sc_ for age and gender and still did not find a difference between glaucoma patients and controls (P = 0.91; footnote to Table 2). Essentially one control was recruited per patient, being the spouse or a neighbor or friend (no consanguinity). An advantage of this approach is that it may control for external factors that influence sleep behavior. A possible drawback is synchronization of the chronotypes of people living together. A small to moderate correlation (0.25–0.40) between chronotypes in husband-wife relationships has been found, which was more the result of assortative mating than caused by cohabitation during marriage [52,53].To explore potential biases related to our recruitment method, we recruited a secondary control group from an independent source. This control group was age and gender matched to the patients and came from the same latitude and longitude. No significant differences in MSF_sc_ were found between the control groups. A strength of this study is that it is the first study that investigated chronotype as a measure of circadian phase in a large group of glaucoma patients, and compared it to controls. We did not screen for the presence of other eye diseases but rather assumed that they would be equally distributed amongst the groups. In this way we aimed for a realistic sample of elderly rather than super normals.

Our results appear to be in agreement with studies on the ipRGC-mediated pupil response, which has repeatedly been found to be similar in early glaucoma compared to controls, while differences did appear in more advanced disease [54–56]. There are several hypotheses why there is no clear difference in chronotype distribution between early and moderate glaucoma patients and controls. First, it is not clear if the ipRGCs disappear in parallel with the image-forming RGCs, or only in advanced disease [57–60]. Second, a lower number of ipRGCs does not necessarily mean less effect—the dose-response curve might be highly nonlinear. A mouse study found that even with the loss of 83% of the ipRGCs, a normal ipRGC-mediated pupil constriction could still be obtained [4]. Moreover, a hamster study reported that the circadian system attained saturation at lower irradiance levels than those required to induce pupil constriction [61]. Interestingly, our results hint towards an increase in the variability of the MSF_sc_ in patients with severe glaucoma, and possibly some advancement of the mean MSF_sc_. If confirmed in other studies, this suggests that some patients have a more advanced sleep phase, with or without a more delayed sleep phase in others. The delay might be explained by the hypothesized change related to the longer than 24-hour intrinsic period. More advanced sleep phases may be explained by some people having an intrinsic period that is shorter than 24 hours and who at the same time suffer from a lack of delaying evening light or miss the acute effects of light keeping them awake [11,62]. An increase in artificial light and the adaptational properties of the non-image forming system might compensate for a change in the MSF_sc_ [63,64]. Whatever the mechanisms involved, individual shifts of the MSF_sc_ to either way will contribute to an increase in variability.

In conclusion, no clear changes were found in the chronotype as determined by sleep phase in patients with glaucoma, especially not in early and moderate glaucoma. In severe glaucoma, chronotype variability seems to increase, possibly alongside some advancement. A more severe loss of ipRGCs in the human retina of glaucoma patients probably results in more difficulties with stable entrainment either due to a reduction in the phase shifting effects of light on the clock or to less influence of light on brain areas directly involved in sleep-wake regulation itself. Future studies might focus on a more in-depth analysis of the circadian clock in severe glaucoma and related disturbance of their quality of life.

## Supporting information

S1 File Data underlying this study. (XLS)
